# Quantifying Anthropogenic Stress on Groundwater Resources

**DOI:** 10.1038/s41598-017-12877-4

**Published:** 2017-10-10

**Authors:** Batool Ashraf, Amir AghaKouchak, Amin Alizadeh, Mohammad Mousavi Baygi, Hamed R. Moftakhari, Ali Mirchi, Hassan Anjileli, Kaveh Madani

**Affiliations:** 1 Department of Civil and Environmental Engineering, University of California, Irvine, USA; 2 Department of Water Engineering, Ferdowsi University of Mashhad, Mashhad, Iran; 3 Department of Civil Engineering and Center for Environmental Resource Management, University of Texas at El Paso, El Paso, USA; 4 Centre for Environmental Policy, Imperial College London, London, UK; 5 Department of Physical Geography, Stockholm University, Stockholm, Sweden

## Abstract

This study explores a general framework for quantifying anthropogenic influences on groundwater budget based on normalized human outflow (h_out_) and inflow (h_in_). The framework is useful for sustainability assessment of groundwater systems and allows investigating the effects of different human water abstraction scenarios on the overall aquifer regime (e.g., depleted, natural flow-dominated, and human flow-dominated). We apply this approach to selected regions in the USA, Germany and Iran to evaluate the current aquifer regime. We subsequently present two scenarios of changes in human water withdrawals and return flow to the system (individually and combined). Results show that approximately one-third of the selected aquifers in the USA, and half of the selected aquifers in Iran are dominated by human activities, while the selected aquifers in Germany are natural flow-dominated. The scenario analysis results also show that reduced human withdrawals could help with regime change in some aquifers. For instance, in two of the selected USA aquifers, a decrease in anthropogenic influences by ~20% may change the condition of depleted regime to natural flow-dominated regime. We specifically highlight a trending threat to the sustainability of groundwater in northwest Iran and California, and the need for more careful assessment and monitoring practices as well as strict regulations to mitigate the negative impacts of groundwater overexploitation.

## Introduction

Global climate change and regional human activities have considerable impacts on the environment and water resources^1,2^, including changes to the hydrological cycle, surface energy budget, and water yield^3–5^. Climate change is considered as one of the main driving forces of change in water availability^6–8^, necessitating adaptation of water resources management, allocation, and operation policies in order to minimize economic ramifications^9–11^. Moreover, dramatic global population growth has contributed to an overall increase in water demands around the world, causing additional anthropogenic stress on water supplies^12^. This phenomenon along with other related factors such as economic and industrial development, land use/cover change, greenhouse gas emissions, and environmental degradation has contributed to the notion of “*anthropogenic drought”* ^13^. The term anthropogenic drought corresponds to human-induced water stress intensified by unsustainable water use relative to the available renewable water. Nearly one third of the world’s population lives under water stress mainly due to unsustainable development^14^.

In many regions, the impacts of increasing water demands are larger than the effects of observed climate change^15^. In the past century, global water demand has increased at more than twice the rate of population growth^16^. Droughts and variability in available water have adversely affected food security, access to safe drinking water, hygiene and public health^17^. Climate change and variability (e.g., changes in snowpack and timing of melt), population growth, overexploitation of groundwater resources and other anthropogenic alterations can further exacerbate water scarcity in the next decades^18^. The compounding effects of climate change and human activities are manifest in the lower latitudes and subtropical regions where temperatures are rising, and potable water resources are being depleted^19^. Numerous studies have documented overuse of scarce water resources worldwide^2,14,20–23^. Projected climate change, increase in future water demands^23^, and expected future population growth, will most likely increase the pressure on available water resources in many countries^2,24–27^.

**Figure 1:**
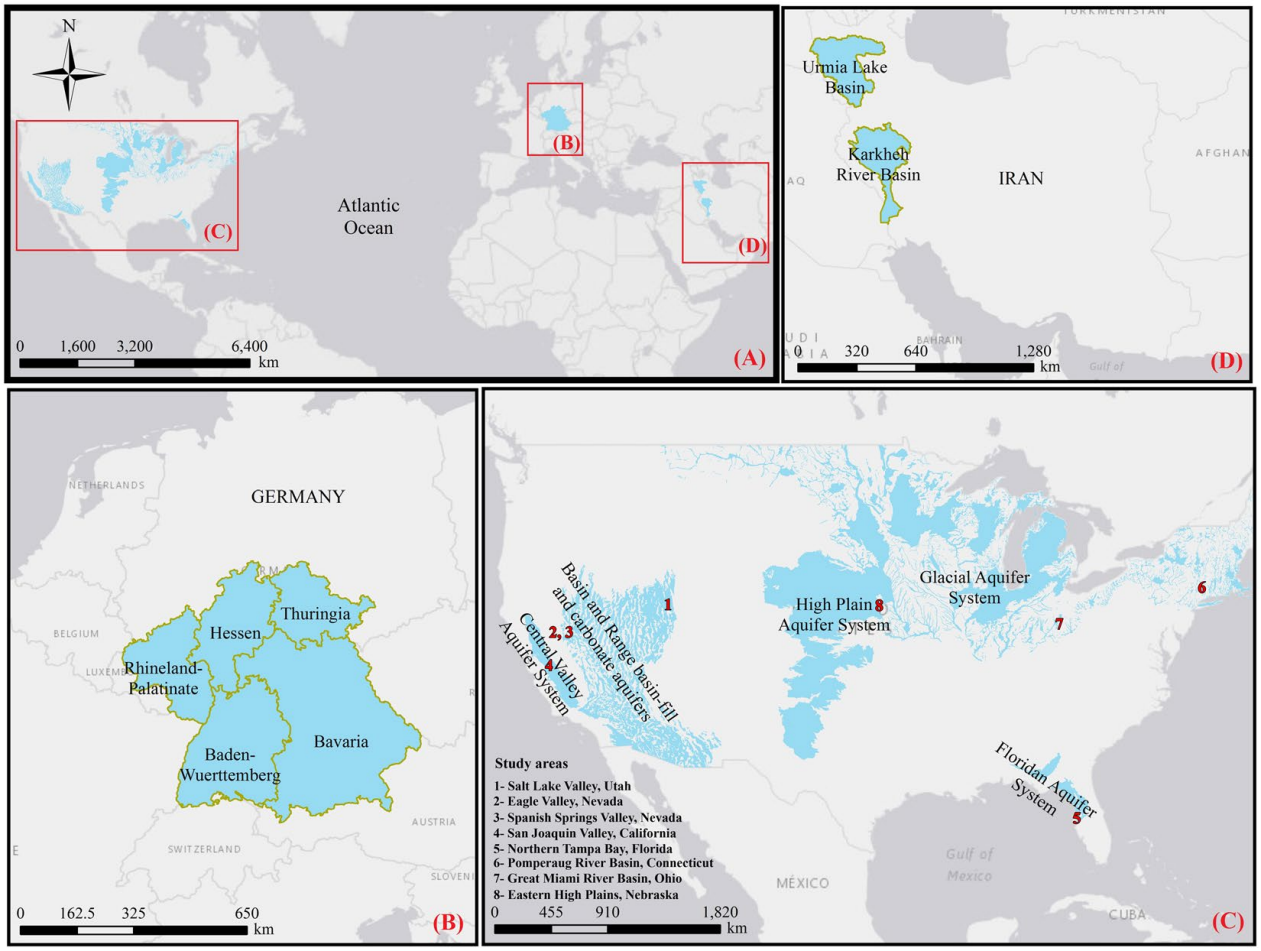
Location of study areas in (**A**) the world (**B**) the Germany, (**C**) the USA and (**D**) the Iran.

Quantifying the impacts of human activities on local hydrological cycle and water yield is essential for improving water management^22,28^. In many parts of the world, groundwater is a major water source that is depleting at an unprecedented rate^29,30^. Several studies have presented frameworks for evaluating changes in groundwater resources^31,32^. Typically, these frameworks are relatively complex and data intensive which limits their practical application. Thus, there is a need for alternative frameworks (e.g. with parsimonious data requirements and less parameters) that not only allows examining the current conditions, but also exploring the effects of different human water withdrawal scenarios on the available resources. This paper aims to offer a better characterization of anthropogenic impacts (i.e., human withdrawal) on groundwater stress and sustainability. The framework quantifies direct human-hydrologic interactions and posed anthropogenic stresses by analyzing the aquifers’ water budget, including human withdrawals and return flows in the governing water-balance equation. We study multiple basins (see Fig. 1) in developed (U.S. and Germany) and developing (Iran) countries and assess water availability under various levels of human water withdrawals (see Materials and Methods, section 4.1). Indeed, this is the first study that compares the water availability in highly-stressed Iranian aquifers with highly managed aquifers located in the U.S. and Germany, drawing insights about the human component and its effect on groundwater regime.

## Results

**Figure 2:**
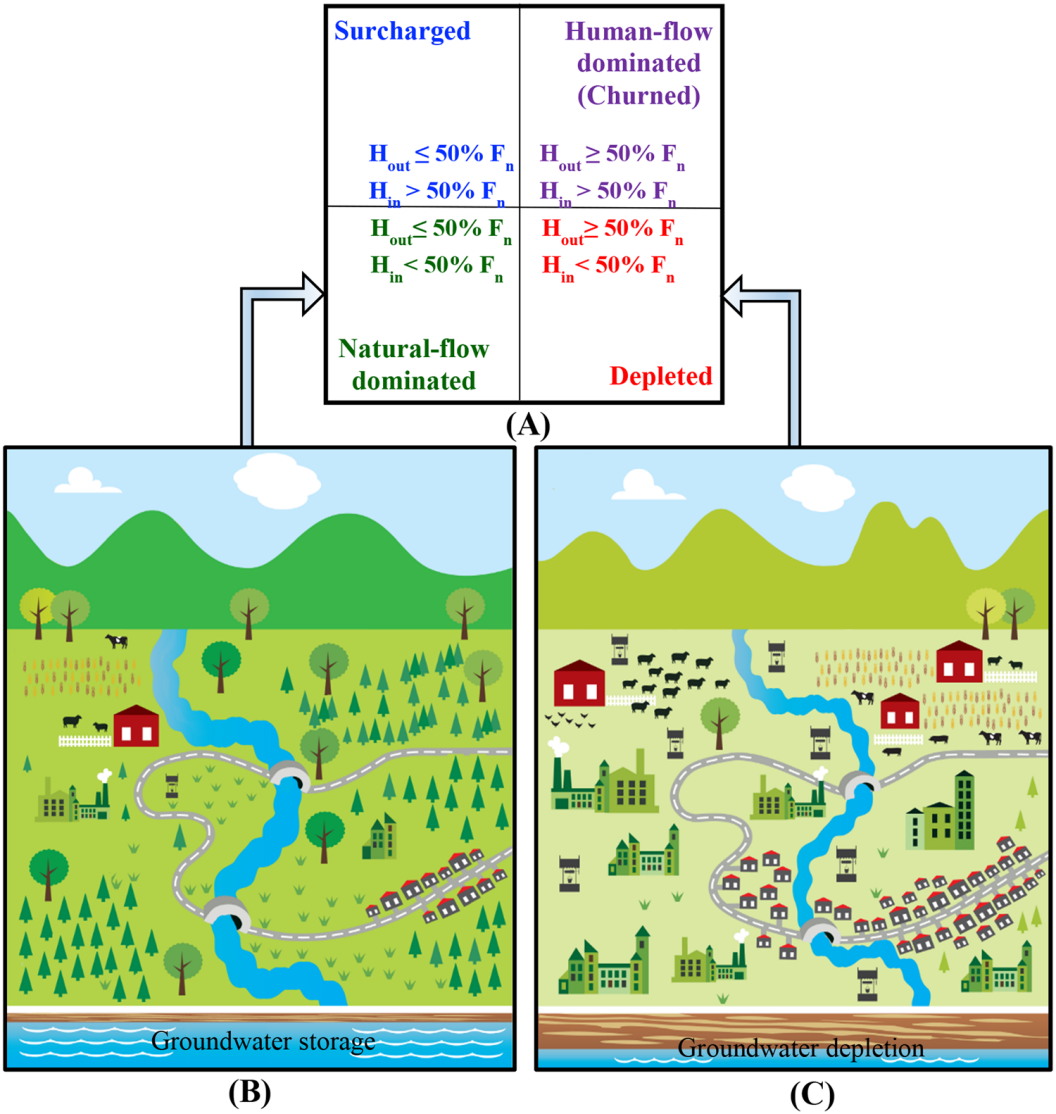
Schematic view of the (**A**) methodology 29,34, (**B**) a system with natural-dominated flow and groundwater storage, and (**C**) a highly developed area with depleted groundwater resources (This figure is created by Jennie Brewton/UCI).

**Figure 3:**
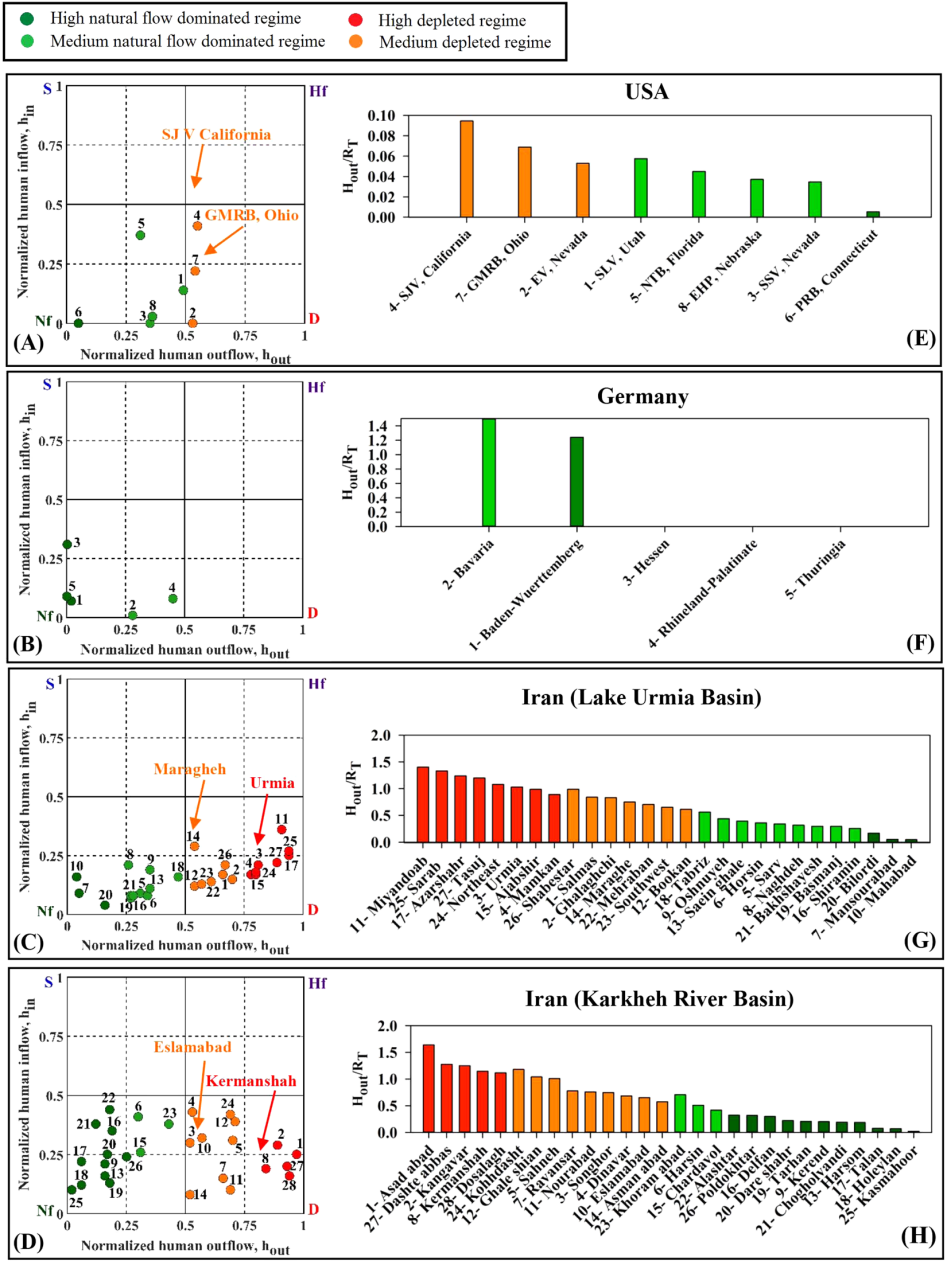
Annual water use regimes (**A**–**D**) and groundwater overdraft ratio (H_out_/R_T_) (**E**–**H**) for selected areas. The color bars are chosen such that they represent the aquifers’ regime type as shown in the left panels (**A**–**D**).

We quantify human-hydrologic interactions by analyzing aquifer water budget including human withdrawals (*H*_*out*_) and return flows (*H*_*in*_) in the governing water-balance equation normalized by the overall net flux of the aquifer based on a framework described by previous studies^28,33^ (see Materials and Methods, Section 4.2). Following this approach, water-use regimes are classified as natural-flow dominated (*Nf*), human-flow dominated (*Hf*) (or churned^28,33^), depleted (*D*), and surcharged (*S*), based on the normalized human water withdrawals and return flows – see Fig. 2A. In this study, groundwater overdraft is determined as a ratio between human withdrawals (H_out_) and recharge (R_T_) in order to evaluate flow condition relative to human water use (see Fig. 2B,C). The left panels in Fig. 3A–D show annual water use regime of the selected study areas in USA, Germany, and Iran. Three out of the eight selected aquifers in the USA have depleted aquifer regime whereas other aquifers are natural-flow dominated (Fig. 3A). It is noteworthy that a natural-flow dominated regime, does not indicate no human activity. It rather implies that the natural flow condition is still dominant. Among all the selected areas in the USA, the San Joaquin Valley (SJV) in California and Pomperaug River Basin (PRB) in Connecticut had the largest and the smallest amounts of human withdrawals (H_out_), respectively. The H_out_ at SJV is 30% larger than the return flow (H_in_). In this aquifer, H_out_ comprised ~55% of net outflow from the aquifer, with natural discharge (D_T_) making up the rest (See Table S1 in Supplementary Materials).

The selected aquifers in Germany have natural-flow dominated regimes, despite substantial human activity and development (Fig. 3B). This is attributed to ample surface water availability and stricter groundwater abstraction regulations relative to the other study areas^34^. These aquifers exhibit samples of water rich basins with relatively healthy aquifers in water availability in an industrialized country with high population density, protected by environmental regulations.

Among the selected aquifers in the Lake Urmia Basin, 15 are depleted while the rest have natural-flow dominated regimes (Fig. 3C). In this basin, the largest and smallest amounts of H_out_ are estimated for Urmia and Mahabad aquifers, respectively. The estimated H_out_ of the Urmia aquifer is four times larger than its H_in_. In this aquifer, H_out_ comprised about 80% of net outflow from the aquifer, with D_T_ making up the rest. In the case of the Mahabad aquifer, H_out_ is only ~4.5% of the net outflow (See Table S3 in Supplementary Material). The estimated values of H_in_ and H_out_ for the presented case studies demonstrate the applicability of the framework to glean insights about the effect of groundwater governance in developing (Iran) and developed (USA and Germany) settings (Tables S1 and S2 in Supplementary Material). In particular, comparing the numbers from Iran with similar aquifers in the USA (in terms of water availability) shows how careless human governance might affect the sustainability of the aquifers.

Figure 3D indicates that half of the 28 studied aquifers in the Karkheh River Basin are of natural-flow dominated type while the rest are depleted. The Asadabad aquifer shows the highest amount of human withdrawals (H_out_) in this basin; almost four times the human return flow (H_in_). This equals 97% of the total net outflow of this aquifer (See Table S4 in Supplementary Material).

The right panels in Fig. 3E–H show the overdraft ratio of the selected study areas. The bar charts illustrate the human withdrawals relative to natural recharge, highlighting the significance of anthropogenic influence in the basins. While substantial amount of water is transferred to SJV, the overall condition is still considered as depleted because of large water withdrawals (Fig. 3A,E, number 4). The lasting depletion may be attributed to further development in the recipient basin due to water transfer-induced perception of water availability which can instigate more withdrawals that, in turn, increase water stress^35,36^.

**Figure 4:**
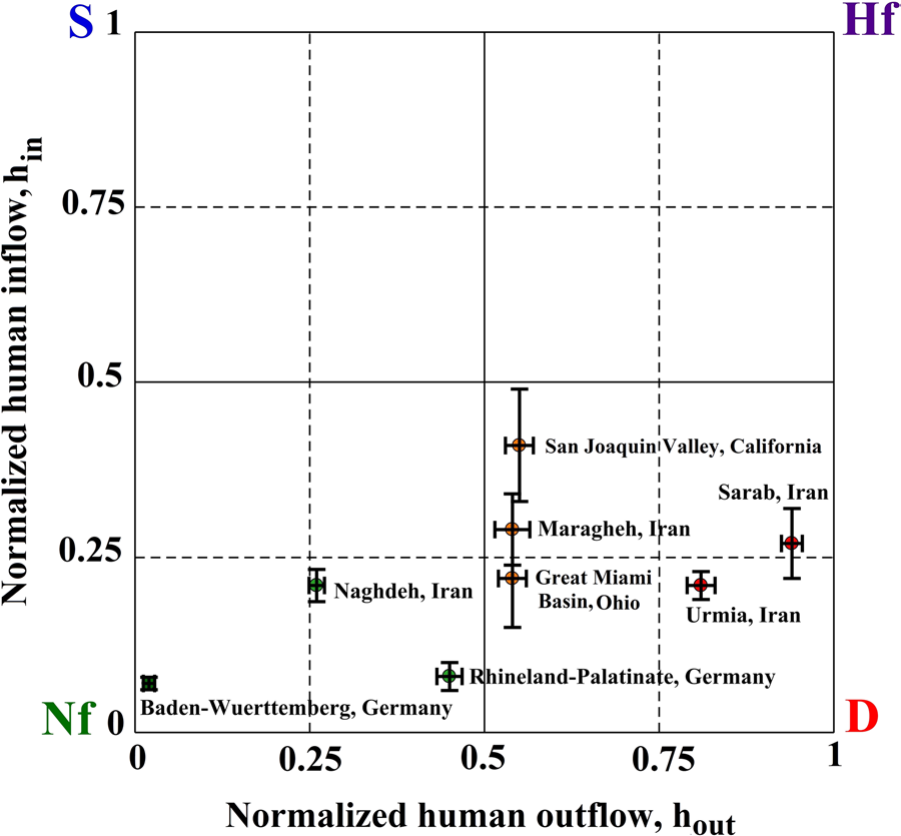
Uncertainty analysis of water use regimes. Error bars show 95% confidence intervals for resulting estimates of h_out_ and h_in_ for some sample aquifers in Fig. 3A–D.

In general, quantification of water use regimes is limited by data availability and uncertainty. Figure 4 shows a set of hypothetical 95% confidence intervals around sample points (h_out_, h_in_) in Fig. 3A–D. The difficulty in distinguishing between different sources of inflow (i.e. human return vs natural) results in greater uncertainties in estimated h_in_ compared to h_out_^33^.

**Figure 5:**
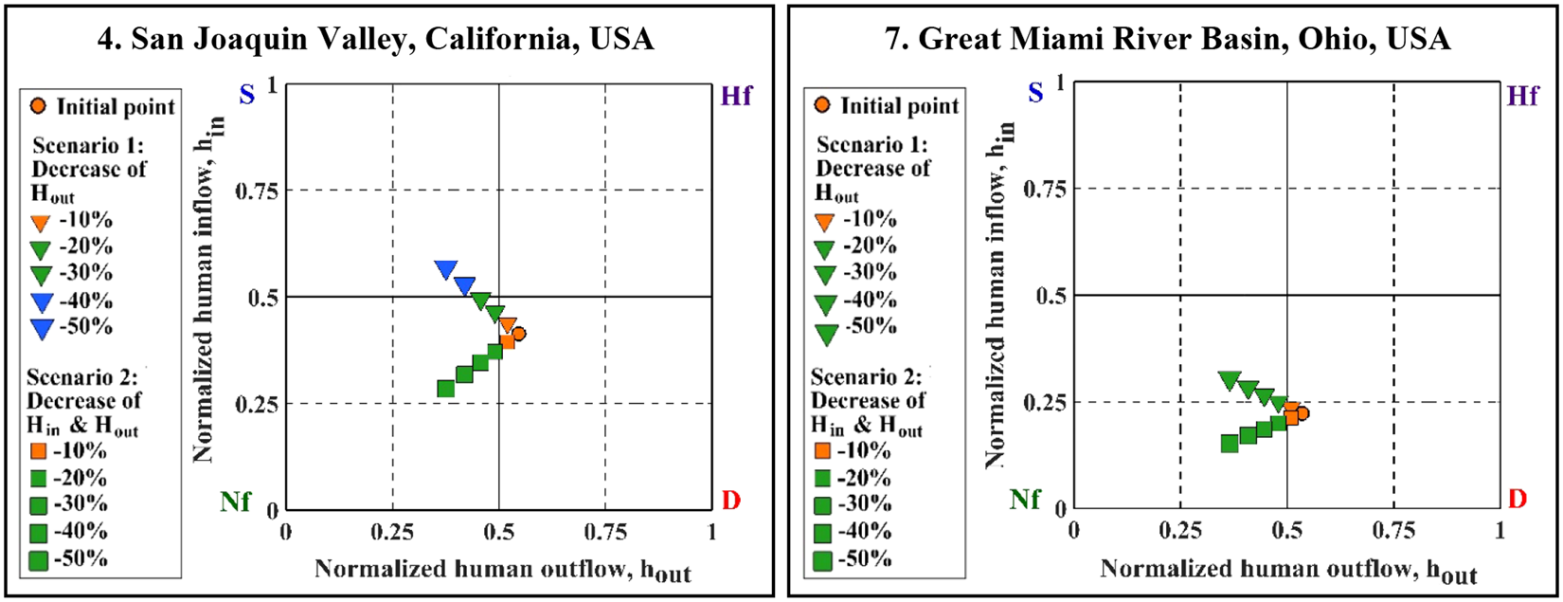
Shifts in annual water use regimes according to two scenarios for two depleted aquifers in USA.

**Figure 6:**
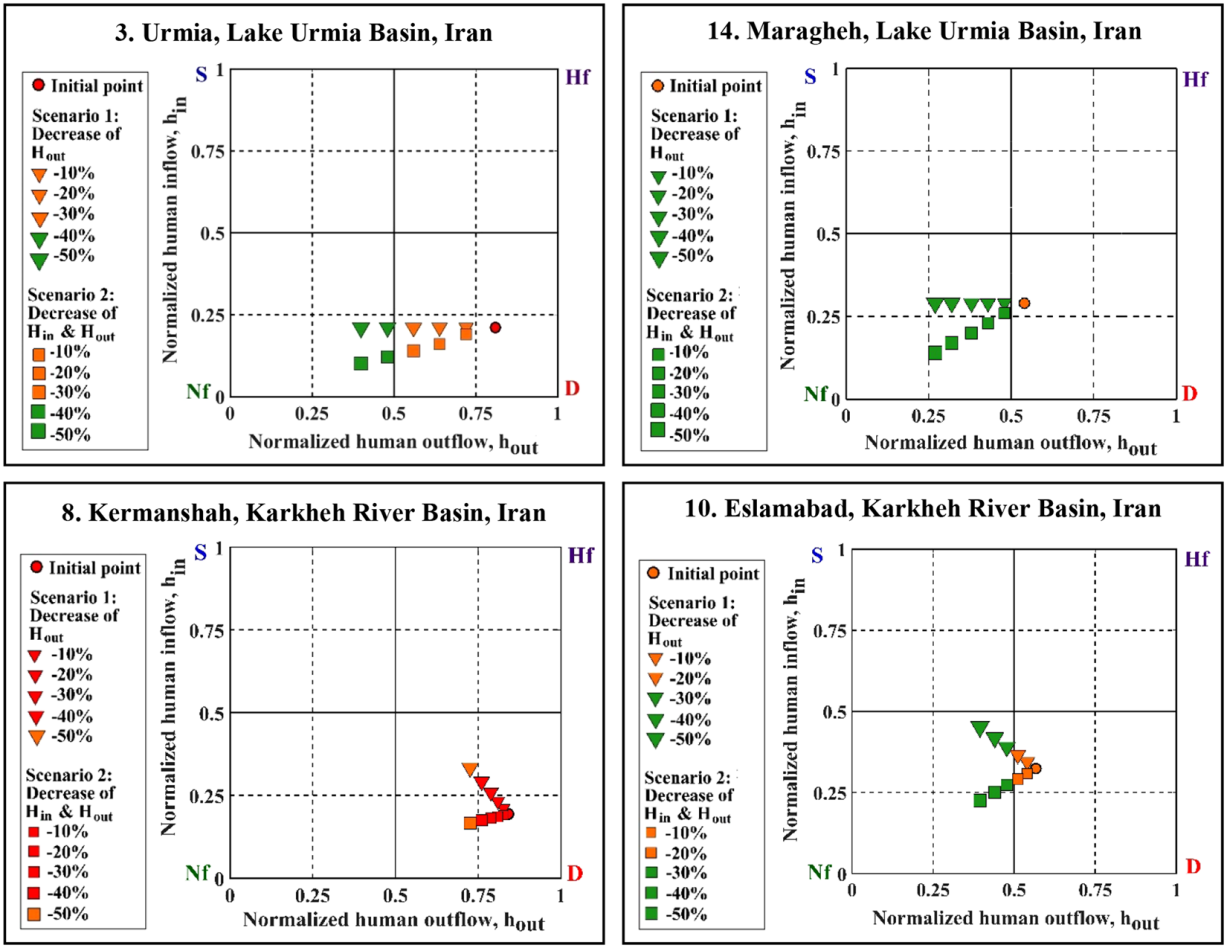
Shifts in annual water use regimes according to two scenarios for four depleted areas in Lake Urmia and Karkheh River Basins, Iran.

Most of the studied aquifers were found to experience significant overdraft as a result of human activities (Fig. 3). Following the characterization of individual aquifer’s regime type, we explore the effects of two scenarios of human influence on selected aquifers (Figs 5 and 6). Hypothetical water management scenarios considered within the context of this water balance approach illustrate the sensitivity of these systems to changes in anthropogenic withdrawals. Scenario 1 only considers a decrease in human withdrawals from the aquifers (H_out_), while Scenario 2 examines decreased H_out_ along with reduced return flow (H_in_). These scenarios represent reductions in H_out_ ranging from 10 to 50% of initial values in each case in order to understand how the aquifer’s regime changes under a broad range of human inflow and outflow conditions. Figures 5 and 6 present examples of aquifers with relatively large H_out_ (depleted regime) out of the selected aquifers in the USA and Iran. The aquifers in Germany are already in the natural-flow dominated regime and have been excluded from scenario analysis.

Figure 5 indicates that reducing the anthropogenic influences (i.e., ~20% decrease in human outflows/inflows) may change the condition of depleted aquifers in the USA to natural-flow dominated. In Great Miami River Basin (GMRB), Ohio, and SJV, California, for example, a 20% decrease in human withdrawals shifts the aquifer regime to natural flow-dominated. In the case of SJV, 40% reduction in H_out_ can even shift a depleted regime to a surcharged regime (see Scenario 1). It is worth pointing out that 20% or 40% reduction in H_out_ is very challenging and might not be practical due to major socio-economic and even political barriers. However, these numbers provide a rough idea about the level of human influence and the resulting deviation from the natural system. Furthermore, we explain below that the explored water withdrawal reduction scenarios do not necessarily lead to a more sustainable hydrologic regime. Likewise, if H_out_ is reduced by ~40%, the Urmia aquifer in the Lake Urmia Basin is expected to shift from depleted regime to natural-flow dominated regime (Fig. 6). For the same impact, a ~10% reduction is required for the Maragheh aquifer in the same basin. Nevertheless, these approximations of reduction rates, with further socio-economic impact assessment studies, can help put the existing challenges of groundwater management into perspective. This may further inform the affected communities about the extent of required resource cutbacks, and the evaluation of potential socio-economic impacts that, ultimately, complicate groundwater table recovery in the foreseeable future.

Reducing human water use does not always improve the aquifer regime condition. In the case of the Kermanshah aquifer, located in the Karkheh River Basin, Iran, H_out_ is significantly larger than the natural recharge (R_T_) (Fig. 6). As such, water use regime remains the same for this aquifer even with large reductions in H_out_. By contrast, Eslamabad aquifer in the same basin is an example of an Iranian aquifer where a 30% reduction in H_out_ can change the regime from depleted to natural-flow dominated. These examples illustrate how the presented approach allows assessing potential changes in the human withdrawals on the overall aquifer condition.

## Discussion

Increasing population along with continuous economic growth made possible by anthropocentric water management and land use/land cover change will aggravate freshwater scarcity by increasing water demand^37,38^. Conjunctive use of surface water and groundwater is commonly practiced around the world to compensate for surface water deficit^39^. It is imperative to evaluate the sustainability of groundwater management because of its crucial importance in providing resilience to buffer the impacts of droughts through relaxing water resource constraints. Our findings, in line with previous works^13,14^, show a trending threat (i.e. Figure 3) to the sustainability of groundwater in northwest Iran and California where natural renewable water supply capacity has been exceeded by water consumption in a significant number of the aquifers. In some parts of Iran, forced migrations have already been observed where farmers lost access to water for irrigation due to excessive surface and groundwater use. As a result of growing anthropogenic droughts, many regions are shifting from water scarcity to *water bankruptcy*, a *de facto* sign of unsustainable development evidenced by severe water stress and associated impacts^27^.

Lake Urmia Basin is an example where scrutiny of aquifer water budget components highlights the significant role of human activities in unsustainable groundwater use. The basin is plagued with anthropocentric water resources development (e.g., dams and diversions) which has triggered an unprecedented decline in Lake’s mean water level. Consequently, the current extent of Lake Urmia is about one-tenth of its original size, creating one of the gravest environmental tragedies in the Middle East^40–43^. Compounding impacts of hydrologic drought and human-induced water deficit caused groundwater storage in the Urmia Basin to decrease at the rate of 11.2 mm/yr during the drought period 2005–2011^44^.

In California, groundwater meets about 30% of the urban and agriculture demand, which can increase to 40% or even higher during drought periods^45^. Reduction in available surface-water during time periods of 1976–77, 1986–92, 2007–09, and 2012–2015 gave rise to groundwater-pumping in the the SJV, which lowered groundwater levels to near or beyond historic lows, and caused aquifer compaction^46^. Land subsidence is a major impact closely related to groundwater depletion, which is already occurring in many groundwater basins across California^47^. This phenomenon has affected more than half of the irrigated agricultural areas in the SJV where some areas subsided up to 9 m by 1970 after nearly half a century of groundwater extraction^48,49^. During the second half of the twentieth century, the SJV’s population has nearly tripled (i.e. from 1.1 million in 1950 to 3.3 million in 2000) and it is projected to exceed 7.3 million by 2040^50^. Furthermore, urbanization in the SJV region is projected to increase by ~133% from ~2.4 million-hectare (Mha) in 2000 to ~5.6 Mha, in 2040^50^. These changes have substantially increased and will further intensify groundwater extraction in SJV, altering the water balance in nearby aquifers. The groundwater depletion in the Central Valley aquifer, has been estimated to be ~60 km^3^ between 1860s and 1961 (~0.6 km^3^/yr) and 80 km^3^ from 1962 to 2003 (~2 km^3^/yr)^51^. This rate reached ~3.1 km^3^/yr between 2003 and 2010 ^20^. These anthropogenic stresses caused the Central Valley aquifer to be identified as one of the hotspots for irrigated agriculture suffering from water depletion in the USA^22^.

Groundwater overdraft can trigger radical changes in future water management practices to cope with water shortages through increasing the pressure on the diminishing resources or additional resource development. Despite various interpretations, in essence, groundwater sustainability refers to the development and use of the resource in a manner that can be maintained for an indefinite time without causing unacceptable environmental, economic, or social consequences^52^. Hence, sustainable groundwater management requires holistic and multi-objective solutions^31^ that aim to minimize the groundwater footprint (a measure of overexploitation) and maintain a balance between i) water withdrawal from the aquifer and ii) aquifer recharge (either naturally or artificially). Further, sustainable groundwater management solutions should maximize the environmental health of the aquifer^53^ without negative impacts on the users’ wellbeing.

Proactive management based on deep regional understanding of water shortage problems and public awareness about anthropogenic droughts and engagement in shared vision watershed planning^54^ is essential for timely demand-side (e.g., conservation and pricing) and supply-side adaptation (e.g., water reuse, inter-basin transfer, desalination, etc.) in order to avoid impatient, myopic remedies whose consequences may be overwhelming^36^.

Adaptive management strategies to re-establish the balance between natural water supply and demand must be informed by rigorous and transparent aquifer sustainability assessments. Extensive outreach programs are required about the links between groundwater depletion and regional vulnerability to and resilience against water scarcity in order to raise public awareness and encourage conservation. This task is complicated by the fact that groundwater availability and withdrawal data are not readily accessible for many regions of the world, especially in developing countries such as Iran. A key step toward this goal would be monitoring the aquifer conditions continuously and adjusting human water use and development goals to avoid groundwater depletion (see Fig. 2A). Finally, a fundamental challenge is to design robust groundwater governance institutions^55^, enforce effective regulatory programs^56^, and provide incentives to restore aquifers in order to ensure aquifer sustainability and long-term economic viability.

Groundwater resources are foundational to resilient societies due to providing the capacity to buffer the impacts of water scarcity by compensating for surface water deficit, especially during droughts. As such, quantifying the impacts of human activities on aquifer systems is essential for sustainable water management. Herein, we presented an approach for evaluation of water budget to facilitate understanding of the entire flow system of the aquifer in order to inform sustainable water resources management. We investigated the impact of human water abstraction on the overall aquifer regime (e.g., depleted, natural flow-dominated, and human flow-dominated) in selected regions in the USA, Germany and Iran. Results illustrate a trending anthropogenic drought threat to groundwater sustainability in northwest Iran and California where water consumption exceeds natural renewable water supply capacity in a significant number of aquifers. This research highlights the need for assessment, monitoring and regulatory practices that effectively mitigate the adverse of anthropogenic withdrawals from groundwater systems within a reasonable timeframe.

## Materials and Methods

### Study locations and data

The selected study areas are located in three geopolitically-disparate regions in North America, Europe and the Middle East. These locations were selected based on availability of water budget data and differences in climate, geology and human water use patterns in order to demonstrate generic applicability of the framework through comparative analysis of groundwater regime. From North America, we considered eight study areas in the USA, encompassing five different aquifers. From Europe, we studied five southern German states with eight different aquifers. Finally, from the Middle East we investigated the Lake Urmia Basin and Karkheh River Basin in western Iran with 27 and 28 aquifers, respectively, which have experienced rapid human development over the past decades (Fig. 1).

We use annual water budget components according to their availability in different time periods. Annual groundwater balance components of the eight areas in the USA from 1997 to 2001 were obtained from the US Geological Survey paper 1737-A^57^. Annual groundwater data for the five German states were obtained from Germany’s Federal Statistical Office from 1998 to 2007. Furthermore, a series of unique data sets each comprising annual groundwater balance components for the aquifers in the Lake Urmia Basin and Karkheh River Basin for the period of 2000–2006 were received from Iran’s Ministry of Energy, providing essential information for evaluating groundwater status analysis in a developing setting alongside developed countries.

### Quantifying human-hydrologic interactions

We use an approach, first developed by [*Weiskel et al*., 2007]^33^, for quantifying direct human-hydrologic interactions by analyzing the aquifers’ water budget including human withdrawals (H_out_) and return flows (H_in_) in the governing water-balance equation^28,33^ as follows:

1 $${R}_{T}+{H}_{in}-ds/dt={D}_{T}+{H}_{out}={F}_{n}$$

where R_T_ is total recharge to the aquifer from precipitation, surface water and adjacent aquifers; D_T_ is total discharge from the aquifer to surface water and adjacent aquifers, as well as evapotranspiration; and ds/dt is change in storage (all the data are from observational dataset). The terms H_in_ and H_out_ denote human inflow (recharge) and outflow (withdrawal). H_in_ and H_out_ can be normalized relative to the net flux (F_n_) as follows^28,33^:

2 $${h}_{in}={H}_{in}/{F}_{n}$$

3 $${h}_{out}={H}_{out}/{F}_{n}$$

Where, h_in_ and h_out_ are normalized human inflow and outflow, respectively.

Based on the values of h_in_ and h_out_ (e.g., above or below average), the water-use regime can be classified as surcharged (S), depleted (D), natural-flow dominated (Nf), and human-flow dominated (Hf) (or churned^28,33^), as shown in the Fig. 2A. Also, water use regimes are defined based on the relationship of human inflows and outflows criteria relative to total flux, and are not functions of the incremental change in storage, so a depleted water use regime may reach a new hydrologic equilibrium. Moreover, all water resources assessment approaches are subject to uncertainty due to various sources of errors (e.g. measurement error, sampling error, model error). We estimated the uncertainty bounds associated with h_in_ (H_in_/D_T_ + H_out_) and hout (H_out_/D_T_ + H_out_) estimates around points for which we had sufficient data using the approach presented by [Weiskel *et al*., 2007] 34. To estimate likelihood intervals for estimated values of two bivariate normal random variables of X = H_out_ or H_in_ and Y = D_T_ + H_out_, we initially assumed a normal probability density function (pdf) for the variables. Then we calculated the pdf of the ratio of these two normal variables (R = X/Y), obtaining the lower and upper likelihood intervals at 95% confidence level.

Groundwater overdraft is defined as the condition of a groundwater basin in which the amount of water withdrawn (H_out_; i.e. via pumping) exceeds the corresponding recharge (R_T_) over a given period of time with no full recovery, even in wet years^58,59^. We determine groundwater overdraft as the ratio between human withdrawals (H_out_) and recharge (R_T_) in order to evaluate the flow condition relative to the human water use (see Fig. 2B,C). Climate change/variability may affect the water availability and consequently yield groundwater overdraft by causing, for example, more frequent hydrologic drought due to altered precipitation pattern/intensity. Moreover, anthropogenic effects (e.g. increased human water use due to population growth, urbanization and land use change) exacerbate the situation and intensify the impacts of the overdraft. Adaptive water resources management nested within holistic resource governance schemes provide opportunities to mitigate the adverse impacts of anthropogenic pressures on groundwater resources.

## Electronic supplementary material

Tables S1, S2, S3, S4
