# Supplementary material for: Quantifying Anthropogenic Stress on Groundwater Resources
Source: Sci Rep. 2017 Oct 10;7:12910. doi: 10.1038/s41598-017-12877-4 (PMC5635107; doi:10.1038/s41598-017-12877-4)
Supplement: Supplementary file 1 — Tables S1, S2, S3, S4 [file 41598_2017_12877_MOESM1_ESM.doc]

[**Scientific Reports**](mailto:scientificreports@nature.com)

Supporting Information for

**[Quantifying Anthropogenic Stress on Groundwater Resources]**

[B. Ashraf ^1,2^, A. AghaKouchak ^1^, A. Alizadeh ^2^, M. Mousavi-Baygi ^2^

H. R. Moftakhari ^1^, A. Mirchi ^3^, H. Anjileli ^1^, K. Madani ^4,5^]

*1- Center of Hydrometeorology and Remote Sensing, University of California, Irvine, USA*

*2-Water Engineering Department, Ferdowsi University of Mashhad, Iran*

*3- Department of Civil Engineering and Center for Environmental Resource Management, University of Texas at El Paso, El Paso, USA*

*4- Centre for Environmental Policy, Imperial College London, UK*

*5- Department of Physical Geography, Stockholm University, Stockholm, Sweden*

Table S1. Annual water budgets of the selected areas in USA.

|  | **Regional study area** | **Area** | **R_T_** | **H_in_** | **Sum of net inflows** | **D_T_** | **H_out_** | **Sum of net outflows** | **Net ds/dt** | **Net flux** | **hin** | **hout** |
| --- | --- | --- | --- | --- | --- | --- | --- | --- | --- | --- | --- | --- |
|  |  | Km^2^ | mm/yr | mm/yr | mm/yr | mm/yr | mm/yr | mm/yr | mm/yr | mm/yr |  |  |
| **1** | Salt Lake Valley (SLV), Utah | 1300 | 243.69 | 39.85 | 283.54 | 144.08 | 140.38 | 284.46 | -0.92 | 284.46 | **0.14** | **0.49** |
| **2** | Eagle Valley (EV), Nevada | 70 | 250.29 | 0 | 250.29 | 117.84 | 132.44 | 250.29 | 0 | 250.29 | **0** | **0.53** |
| **3** | Spanish Springs Valley (SSV), Nevada | 15.6 | 350.96 | 0 | 350.96 | 229.29 | 121.67 | 350.96 | 0.00 | 350.96 | **0** | **0.35** |
| **4** | San Joaquin Valley (SJV), California | 2700 | 429.62 | 307.95 | 737.57 | 337.42 | 406.37 | 743.79 | -6.22 | 743.79 | **0.41** | **0.55** |
| **5** | Northern Tampa Bay (NTB), Florida | 5426 | 271.16 | 146.17 | 417.34 | 275.40 | 121.76 | 397.15 | 20.18 | 397.15 | **0.37** | **0.31** |
| **6** | Pomperaug River Basin (PRB), Connecticut | 128 | 378.97 | 0.00 | 378.97 | 358.73 | 20.25 | 378.97 | 0.00 | 378.97 | **0.00** | **0.05** |
| **7** | Great Miami River Basin (GMRB), Ohio | 1700 | 167.69 | 48.09 | 215.78 | 100.29 | 115.51 | 215.80 | -0.02 | 215.80 | **0.22** | **0.54** |
| **8** | Eastern High Plains (EHP), Nebraska | 389 | 453.82 | 14.88 | 468.70 | 300.42 | 168.25 | 468.67 | 0.03 | 468.67 | **0.03** | **0.36** |

Table S2. Annual water budgets of the selected areas in Germany.

|  | **Regional study area** | **Area** | **R_T_** | **H_in_** | **Sum of net inflows** | **D_T_** | **H_out_** | **Sum of net outflows** | **Net ds/dt** | **Net flux** | **hin** | **hout** |
| --- | --- | --- | --- | --- | --- | --- | --- | --- | --- | --- | --- | --- |
|  |  | Km^2^ | mm/yr | mm/yr | mm/yr | mm/yr | mm/yr | mm/yr | mm/yr | mm/yr |  |  |
| **1** | Baden-Wuerttemberg | 141.69 | 132.26 | 63.74 | 196 | 933.01 | 16.41 | 949.42 | -753.42 | 949.42 | **0.07** | **0.02** |
| **2** | Bavaria | 143.78 | 213.59 | 16.32 | 229.91 | 1088.28 | 417.46 | 1505.74 | -1275.83 | 1505.74 | **0.01** | **0.28** |
| **3** | Hessen | 155.08 | 2478.08 | 90.3 | 2568.38 | 291.26 | 0.46 | 291.72 | 2276.66 | 291.72 | **0.31** | **0** |
| **4** | Rhineland-Palatinate | 20 | 0 | 238.05 | 238.05 | 1625.65 | 1325.75 | 2978.4 | -2740.35 | 2978.4 | **0.08** | **0.45** |
| **5** | Thuringia | 21.16 | 0 | 72.78 | 72.78 | 850.8 | 0 | 850.8 | -778.02 | 850.8 | **0.09** | **0** |

Table S3. Annual water budgets of the selected areas in Urmia lake Basin of Iran.

|  | **Regional study area** | **Area** | **R_T_** | **H_in_** | **Sum of net inflows** | **D_T_** | **H_out_** | **Sum of net outflows** | **Net ds/dt** | **Net flux** | **hin** | **hout** |
| --- | --- | --- | --- | --- | --- | --- | --- | --- | --- | --- | --- | --- |
|  |  | Km^2^ | mm/yr | mm/yr | mm/yr | mm/yr | mm/yr | mm/yr | mm/yr | mm/yr |  |  |
| **1** | Salmas | 293.35 | 500.29 | 111.15 | 611.44 | 215.17 | 420.86 | 636.03 | -24.6 | 636.03 | **0.17** | **0.66** |
| **2** | Ghalaghchi | 9.28 | 281.25 | 49.89 | 331.14 | 99.14 | 233.84 | 332.97 | -1.83 | 332.97 | **0.15** | **0.7** |
| **3** | Urmia | 682.88 | 618.01 | 162.94 | 780.95 | 154.39 | 637.36 | 791.75 | -10.8 | 791.75 | **0.21** | **0.81** |
| **4** | Mamkan | 25.68 | 226.64 | 43.46 | 270.09 | 55.69 | 201.71 | 257.4 | 12.69 | 257.4 | **0.17** | **0.78** |
| **5** | Sarv | 43.68 | 692.31 | 65.51 | 757.82 | 515.57 | 236.49 | 752.06 | 5.76 | 752.06 | **0.09** | **0.31** |
| **6** | Horsin | 20.14 | 307.85 | 26.32 | 334.16 | 218.97 | 111.72 | 330.69 | 3.48 | 330.69 | **0.08** | **0.34** |
| **7** | Mansourabad | 52.01 | 449.14 | 43.43 | 492.58 | 459.72 | 23.84 | 483.56 | 9.02 | 483.56 | **0.09** | **0.05** |
| **8** | Naghdeh | 228.37 | 232.91 | 58.95 | 291.86 | 205.41 | 73.83 | 279.24 | 12.62 | 279.24 | **0.21** | **0.26** |
| **9** | Oshnuyeh | 95.93 | 520.28 | 123.27 | 643.54 | 426.46 | 228.29 | 654.75 | -11.21 | 654.75 | **0.19** | **0.35** |
| **10** | Mahabad | 78.95 | 215.83 | 42.46 | 258.29 | 247.5 | 10.89 | 258.39 | -0.1 | 258.39 | **0.16** | **0.04** |
| **11** | Miyandoab | 852.60 | 170 | 95.07 | 265.07 | 24.31 | 238.15 | 262.46 | 2.6 | 262.46 | **0.36** | **0.91** |
| **12** | Bookan | 75.10 | 588.02 | 76.84 | 664.86 | 305.06 | 360.19 | 665.25 | -0.39 | 665.25 | **0.12** | **0.54** |
| **13** | Saein ghale | 162.13 | 653.55 | 80.03 | 733.58 | 483.75 | 257.08 | 740.83 | -7.25 | 740.83 | **0.11** | **0.35** |
| **14** | Maraghe | 223.13 | 217.99 | 87.24 | 305.23 | 140.19 | 163.49 | 303.68 | 1.55 | 303.68 | **0.29** | **0.54** |
| **15** | Ajabshir | 61.62 | 455.53 | 95.82 | 551.36 | 110.03 | 449.37 | 559.4 | -8.04 | 559.4 | **0.17** | **0.8** |
| **16** | Shiramin | 18.26 | 226.73 | 14.9 | 241.62 | 161.01 | 58.05 | 219.06 | 22.56 | 219.06 | **0.07** | **0.27** |
| **17** | Azarshahr | 127.46 | 448.69 | 149.43 | 598.12 | 37.11 | 556.17 | 593.28 | 4.83 | 593.28 | **0.25** | **0.94** |
| **18** | Tabriz | 575.99 | 197.54 | 37.97 | 235.51 | 123.53 | 111.29 | 234.81 | 0.7 | 234.81 | **0.16** | **0.47** |
| **19** | Basmanj | 42.73 | 653.41 | 56.62 | 710.03 | 516.97 | 193.07 | 710.04 | -0.01 | 710.04 | **0.08** | **0.27** |
| **20** | Bilordi | 40.62 | 837.52 | 37.63 | 875.15 | 736.83 | 141.06 | 877.89 | -2.74 | 877.89 | **0.04** | **0.16** |
| **21** | Bakhshayesh | 146.54 | 163.71 | 13.89 | 177.6 | 123.17 | 48.45 | 171.63 | 5.97 | 171.63 | **0.08** | **0.28** |
| **22** | Mehraban | 82.59 | 326.67 | 52.31 | 378.98 | 149.78 | 230.05 | 379.83 | -0.85 | 379.83 | **0.14** | **0.61** |
| **23** | Southwest | 30.03 | 318.02 | 46.55 | 364.57 | 154.85 | 207.46 | 362.3 | 2.26 | 362.3 | **0.13** | **0.57** |
| **24** | Northeast | 80.67 | 165.37 | 39.79 | 205.16 | 44.25 | 178.51 | 222.76 | -17.6 | 222.76 | **0.18** | **0.8** |
| **25** | Sarab | 460.86 | 121.62 | 47.43 | 169.05 | 10.76 | 161.78 | 172.55 | -3.49 | 172.55 | **0.27** | **0.94** |
| **26** | Shabestar | 436.43 | 152.37 | 47.25 | 199.62 | 72.93 | 150.68 | 223.61 | -23.99 | 223.61 | **0.21** | **0.67** |
| **27** | Tasuj | 190.35 | 264.93 | 77.91 | 342.84 | 40.35 | 317.21 | 357.55 | -14.71 | 357.55 | **0.22** | **0.89** |

Table S4. Annual water budgets of the selected areas in Karkheh River Basin of Iran.

|  | **Regional study area** | **Area** | **R_T_** | **H_in_** | **Sum of net inflows** | **D_T_** | **H_out_** | **Sum of net outflows** | **Net ds/dt** | **Net flux** | **hin** | **hout** |
| --- | --- | --- | --- | --- | --- | --- | --- | --- | --- | --- | --- | --- |
|  |  | Km^2^ | mm/yr | mm/yr | mm/yr | mm/yr | mm/yr | mm/yr | mm/yr | mm/yr |  |  |
| **1** | Asad abad | 482 | 271.99 | 114.94 | 386.93 | 14.52 | 446.06 | 460.58 | -73.65 | 460.58 | **0.25** | **0.97** |
| **2** | Kangavar | 535 | 133.27 | 54.21 | 187.48 | 19.81 | 166.73 | 186.54 | 0.93 | 186.54 | **0.29** | **0.89** |
| **3** | Songhor | 357 | 104.48 | 44.54 | 149.02 | 70.87 | 78.15 | 149.02 | 0.00 | 149.02 | **0.30** | **0.52** |
| **4** | Dinavar | 229 | 182.53 | 100.44 | 282.97 | 110.04 | 124.89 | 234.93 | 48.03 | 234.93 | **0.43** | **0.53** |
| **5** | Sahneh | 656 | 130.34 | 58.69 | 189.02 | 57.47 | 131.55 | 189.02 | 0.00 | 189.02 | **0.31** | **0.70** |
| **6** | Harsin | 71 | 140.85 | 97.18 | 238.03 | 167.61 | 71.83 | 239.44 | -1.41 | 239.44 | **0.41** | **0.30** |
| **7** | Ravansar | 1463 | 88.86 | 15.86 | 104.72 | 35.48 | 69.24 | 104.72 | 0.00 | 104.72 | **0.15** | **0.66** |
| **8** | Kermanshah | 984 | 152.74 | 40.35 | 193.09 | 33.03 | 175.30 | 208.33 | -15.24 | 208.33 | **0.19** | **0.84** |
| **9** | Kerend | 89 | 88.76 | 23.60 | 112.36 | 94.38 | 17.98 | 112.36 | 0.00 | 112.36 | **0.21** | **0.16** |
| **10** | Eslamabad | 562 | 81.32 | 30.43 | 111.74 | 40.75 | 53.20 | 93.95 | 17.79 | 93.95 | **0.32** | **0.57** |
| **11** | Nourabad | 116 | 93.97 | 10.34 | 104.31 | 32.76 | 71.55 | 104.31 | 0.00 | 104.31 | **0.10** | **0.69** |
| **12** | Ghale shian | 115 | 83.48 | 47.83 | 131.30 | 35.65 | 86.96 | 122.61 | 8.70 | 122.61 | **0.39** | **0.71** |
| **13** | Harsom | 279 | 48.03 | 9.32 | 57.35 | 48.39 | 8.96 | 57.35 | 0.00 | 57.35 | **0.16** | **0.16** |
| **14** | Asman abad | 50 | 94.00 | 8.00 | 102.00 | 50.00 | 54.00 | 104.00 | -2.00 | 104.00 | **0.08** | **0.52** |
| **15** | Chardavol | 138 | 178.26 | 63.04 | 241.30 | 166.67 | 74.64 | 241.30 | 0.00 | 241.30 | **0.26** | **0.31** |
| **16** | Delfan | 454 | 96.92 | 52.20 | 149.12 | 120.04 | 29.07 | 149.12 | 0.00 | 149.12 | **0.35** | **0.19** |
| **17** | Talan | 153 | 33.99 | 9.80 | 43.79 | 41.18 | 2.61 | 43.79 | 0.00 | 43.79 | **0.22** | **0.06** |
| **18** | Holeylan | 467 | 83.51 | 11.13 | 94.65 | 86.51 | 6.00 | 92.51 | 2.14 | 92.51 | **0.12** | **0.06** |
| **19** | Tarhan | 216 | 53.24 | 7.87 | 61.11 | 50.00 | 11.11 | 61.11 | 0.00 | 61.11 | **0.13** | **0.18** |
| **20** | Dare shahr | 243 | 150.21 | 50.21 | 200.41 | 166.26 | 33.74 | 200.00 | 0.41 | 200.00 | **0.25** | **0.17** |
| **21** | Chogholvandi | 423 | 80.38 | 48.46 | 128.84 | 113.48 | 15.37 | 128.84 | 0.00 | 128.84 | **0.38** | **0.12** |
| **22** | Alashtar | 511 | 70.84 | 56.56 | 127.40 | 104.50 | 22.90 | 127.40 | 0.00 | 127.40 | **0.44** | **0.18** |
| **23** | Khoram abad | 524 | 90.46 | 57.44 | 147.90 | 85.88 | 63.93 | 149.81 | -1.91 | 149.81 | **0.38** | **0.43** |
| **24** | Kohhdasht | 593 | 41.99 | 30.35 | 72.34 | 22.77 | 49.58 | 72.34 | 0.00 | 72.34 | **0.42** | **0.69** |
| **25** | Kasmahoor | 81 | 56.79 | 6.17 | 62.96 | 61.73 | 1.23 | 62.96 | 0.00 | 62.96 | **0.10** | **0.02** |
| **26** | Poldokhtar | 113 | 206.19 | 64.60 | 270.80 | 204.42 | 66.37 | 270.80 | 0.00 | 270.80 | **0.24** | **0.25** |
| **27** | Dashte abbas | 499 | 182.97 | 49.90 | 232.87 | 16.23 | 233.47 | 249.70 | -16.83 | 249.70 | **0.20** | **0.93** |
| **28** | Dosalagh | 497 | 138.03 | 25.75 | 163.78 | 9.66 | 154.12 | 163.78 | 0.00 | 163.78 | **0.16** | **0.94** |
